# Association between *XPF* Polymorphisms and Cancer Risk: A Meta-Analysis

**DOI:** 10.1371/journal.pone.0038606

**Published:** 2012-07-02

**Authors:** Ting-Yan Shi, Jing He, Li-Xin Qiu, Mei-Ling Zhu, Meng-Yun Wang, Xiao-Yan Zhou, Jiali Han, Hongpin Yu, Rong-Yu Zang, Qingyi Wei

**Affiliations:** 1 Cancer Research Laboratory, Fudan University Shanghai Cancer Center, Shanghai, China; 2 Department of Oncology, Shanghai Medical College, Fudan University, Shanghai, China; 3 Department of Medical Oncology, Fudan University Shanghai Cancer Center, Shanghai, China; 4 Department of Gynecologic Oncology, Fudan University Shanghai Cancer Center, Shanghai, China; 5 Department of Dermatology, Brigham and Women’s Hospital, Harvard Medical School, Boston, Massachusetts, United States of America; 6 Channing Laboratory, Department of Medicine, Brigham and Women’s Hospital and Harvard Medical School, Boston, Massachusetts, United States of America; 7 Department of Epidemiology, Harvard School of Public Health, Boston, Massachusetts, United States of America; 8 Department of Epidemiology, The University of Texas M.D. Anderson Cancer Center, Houston, Texas, United States of America; University of Ottawa, Canada

## Abstract

**Background:**

Xeroderma pigmentosum complementation group F (*XPF* or *ERCC4*) plays a key role in DNA repair that protects against genetic instability and carcinogenesis. A series of epidemiological studies have examined associations between *XPF* polymorphisms and cancer risk, but the findings remain inconclusive.

**Methodology/Principal Findings:**

In this meta-analysis of 47,639 cancer cases and 51,915 controls, by searching three electronic databases (i.e., MEDLINE, EMBASE and CNKI), we summarized 43 case-control studies from 29 publications on four commonly studied polymorphisms of *XPF* (i.e., rs1800067, rs1799801, rs2020955 and rs744154), and we did not find statistical evidence of any significant association with overall cancer risk. However, in stratification analyses, we found a significant association of *XPF*-rs1799801 with a reduced cancer risk in Caucasian populations (4,845 cases and 5,556 controls; recessive model: OR = 0.87, 95% CI = 0.76–1.00, *P* = 0.049, *P* = 0.723 for heterogeneity test, *I^2^* = 0). Further genotype-phenotype correlation analysis showed that the homozygous variant CC genotype carriers had higher *XPF* expression levels than that of the TT genotype carriers (Student’s *t* test for a recessive model: *P* = 0.046). No publication bias was found by using the funnel plot and Egger’s test.

**Conclusion:**

This meta-analysis suggests a lack of statistical evidence for the association between the four *XPF* SNPs and overall risk of cancers. However, *XPF*-rs1799801 may be associated with cancer risk in Caucasian populations, which needs to be further validated in single large, well-designed prospective studies.

## Introduction

**Figure 1:**
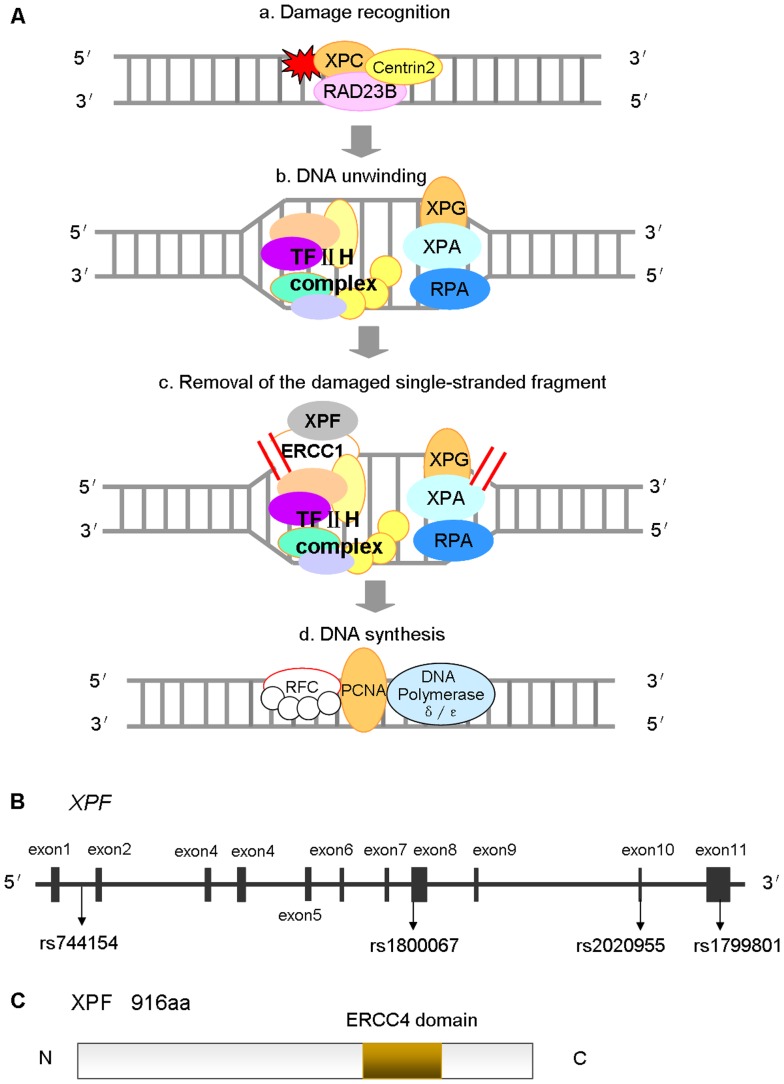
Structural characteristics and function of *XPF* as modified from KEGG and GeneBank database. (A) NER involves at least four steps: (a) damage recognition by a complex of bound proteins including XPC, (b) unwinding of the DNA by the TFIIH complex that includes XPD, (c) removal of the damaged single-stranded fragment by molecules including an ERCC1/XPF complex, and (d) synthesis by DNA polymerases; (B) The *XPF* gene map labeled with 11 exons, and four polymorphisms that have been commonly studied for their associations with cancer risk (i.e., rs1800067, rs1799801, rs2020955 and rs744154); (C) The XPF protein consists of 916 amino acids, containing an ERCC4 domain. Abbreviation: NER, nucleotide excision repair; KEGG, Kyoto Encyclopedia of Genes and Genomes.

Nucleotide excision repair (NER) is the most versatile, well studied DNA repair mechanism in humans, mainly responsible for repairing bulky DNA damage, such as DNA adducts caused by UV radiation, mutagenic chemicals, or chemotherapeutic drugs [1]. The repair process includes excising and removing damaged nucleotides and synthesizing to fill the resultant gap by using the complementary DNA strand as a template [1]. Therefore, reduced DNA repair capacity (DRC) may lead to genomic instability and carcinogenesis, and genes involved in the NER pathway are candidate cancer susceptibility genes [1]–[3]. NER involves at least four steps (**Figure 1A**): (a) damage recognition by a complex of bound proteins including XPC; (b) unwinding of the DNA by the TFIIH complex that includes XPD; (c) removal of the damaged single-stranded fragment by molecules including an ERCC1/XPF complex; and (d) synthesis by DNA polymerases [4].

One of the NER genes, xeroderma pigmentosum complementation group F (*XPF*), also called excision repair cross-complimentary group 4 (*ERCC4*), is located on chromosome 16p13.12, contains 11 exons and spans approximately 28.2 kb (**Figure 1B**) [5]. It is a key component involved in the 5′ incision made during NER [2]. The XPF protein consists of 916 amino acids, containing an ERCC4 domain (**Figure 1C**) that is one of the nuclease family, in which essential meiotic endonuclease 1 (EME1) acts as an essential component of a Holliday junction resolvase to interact with MUS81 [6], [7]. The ERCC4 domain is also necessary for forming a tight complex with ERCC1 as a structure-specific DNA repair endonuclease responsible for the 5′-primer incision during DNA excision repair (**Figure 1C**) [8], [9]. In addition to NER, this complex is suggested to play a role in removal of DNA interstrand cross-links (ICL) [10] and DNA double-strand breaks (DSB) as well [11].

To date, a total of 580 single nucleotide polymorphisms (SNPs) in the *XPF* gene have been reported according to the dbSNP database (http://www.ncbi.nlm.nih.gov/projects/SNP/snp_ref.cgi?chooseRs=all&go=Go&locusId=2072), some of which have been shown as susceptibility loci for several kinds of cancer, including those of the breast, endometrium, and colorectum [12]–[15]. For example, an important and frequent *XPF* polymorphism – rs1800067 (Arg415Gln), which results in an arginine-to-glutamine transition at codon 415 (**Figure 1B**), may affect protein interactions, diminish the activity of the ERCC1/XPF complex and alter genetic susceptibility to cancer [16]. The *XPF*-rs1799801 (Ser835Ser) polymorphism (**Figure 1B**), though not altering amino acids, was reported to be a risk factor for cancer [17]. Another commonly studied *XPF* SNP, (rs2020955) is a serine-to-proline transition at codon 662, which is less frequent but potentially affecting the function of the gene. Interestingly, another commonly studied *XPF* SNP (rs744154) is located at intron 1, and its functionality is unknown (**Figure 1B**). To date, associations of these four SNPs with cancer risk have been investigated by a number of reported studies [12]–[15], [17]–[41], but the results are inconclusive, partially because of a possible weak effect of the polymorphisms on cancer risk or study design with a relatively small sample size to detect such weak associations in each of the published studies. Therefore, we performed a meta-analysis that assemblies a large sample size to derive a more precise risk estimate for the commonly studied *XPF* polymorphisms (each investigated at least by four published studies) with an improved statistic power to detect their associations with cancer risk.

## Methods

### Literature Search Strategy

We first used two electronic databases (MEDLINE and EMBASE) to identify all case-control studies published to date on an association between *XPF* polymorphisms and cancer risk (the last search update on December 16, 2011, using the search terms “*XPF*” or “*ERCC4*”; “cancer”, “neoplasia”, “malignancy” or “carcinoma”; “polymorphism” or “variant”). To expand the coverage of our searches, we further searched Chinese National Knowledge Infrastructure (CNKI) database (http://www.cnki.net) (1979–), using the terms “*XPF*” or “*ERCC4*”; “cancer” in Chinese. Additional published studies on this topic in the references of each publication were also hand reviewed. We further contacted study investigators to identify some unpublished or submitted studies. Only studies with a full text article were included. The authors of published papers were also contacted directly, if crucial data were not reported in original papers. When more than one of the same patient populations were included in different publications, only the most recent or complete study with the largest sample size was included in this meta-analysis.

### Selection Criteria

Studies included in the current meta-analysis had to meet the following criteria: evaluation of *XPF* polymorphisms and cancer risk; more than three studies were available for a certain SNP; written in English or Chinese; case-control study design; sufficient information needed to estimate odds ratios (ORs) and their 95% confidence intervals (CIs); and independent from other studies to avoid double weighting in the estimates derived from the same study. In addition, investigations in control subjects with cancer patients or departure from Hardy-Weinberg equilibrium (HWE) were also excluded from the final analysis.

### Data Extraction

Two authors (STY and HJ) independently extracted data and reached a consensus on all of the items. The following information was extracted from each report: the first author, year of publication, country of origin, ethnicity, cancer type, study type (retrospective and prospective), control source [population-based (PB), hospital-based (HB) and family-based (FB)], DNA source (e.g., blood, lymphocytes, and buccal cells), and genotyping methods, total numbers of cases and controls, minor allele frequency (MAF) and numbers of cases and controls with the wild-type, heterozygous and homozygous genotypes. For studies including subjects of different racial descents and having complete genotyping data for each race, data were extracted separately for each ethnic group (categorized as Caucasian, African American, Asian or others). When a study did not state the detailed genotyping result for each ethnic group or if it was impossible to separate participants according to the data presented, the sample was termed as “mixed”. If the numbers of genotyping methods in a study were more than three and no detailed method information was given, the methods were defined “pooled”. Furthermore, references involving different ethnic groups, different types of cancer and different institutions were divided into multiple single study samples for subgroup analyses.

### Quantitative Data Synthesis

The numbers of cases and controls by the wild-type, heterozygous and homozygous genotypes were collected from each study to evaluate the risk of developing cancers (ORs and 95% CIs). We further performed stratification analyses by cancer type (if one cancer type was investigated in less than three studies, it would be merged into the “other cancers” group), study type (retrospective and prospective), ethnicity (Caucasian, African American, Asian or others), control source (HB, PB and FB) and sample size (numbers of cases <500, 500–1000 and >1000).

HWE was evaluated for control subjects of each study, using the goodness-of-fit χ^2^-test, and *P*<0.05 was considered representative of departure from HWE. Crude ORs with 95% CIs were used to assess the strength of associations between the *XPF* polymorphisms and cancer risk. The pooled ORs were calculated by using homozygous model (variant homozygous *vs.* wild-type) and recessive model (homozygous *vs.* heterozygous + wild-type). For each study, we estimated statistical power to detect an OR of 1.50 (for a risk effect) or its reciprocal 0.67 (for a protective effect), with an α level equal to the observed *P* value [42]. The χ^2^-based Q test was performed to assess between-study heterogeneity and considered significant if *P*<0.05 [43].

Heterogeneity was also quantified with the *I^2^* statistic, a value that indicates what proportion of the total variation across studies is beyond chance. Specifically, 0% indicates no observed heterogeneity, and larger values show increasing heterogeneity [44]. When *P* value of the heterogeneity test was ≥0.05, the fixed-effects model, based on the Mantel-Haenszel method was used, which assumes the same homogeneity of effect size across all studies [45]. Otherwise, the random-effects model, based on the DerSimonian and Laird method, was more appropriate, which tends to provide wider 95% CIs as the results of the constituent studies differ among themselves [46]. Subgroup analyses were also performed by cancer type, ethnicity, control source and sample size. To assess the effects of individual studies on the overall risk of cancer, sensitivity analysis was performed by excluding each study at a time individually and recalculating the ORs and 95% CIs. Potential publication bias was estimated by the inverted funnel plot, in which the standard error of log (OR) of each study was plotted against its log (OR) [47], and an asymmetric plot suggests a possible publication bias. Funnel plot asymmetry was assessed by the method of Egger’s linear regression test, a linear regression approach to measure funnel plot asymmetry on the natural logarithm scale of the ORs [47]. The significance of the intercept was determined by the *t* test as suggested by Egger, and *P*<0.05 was considered representative of statistically significant publication bias [47]. If publication bias existed, the Duval and Tweedie nonparametric “trim and fill” method was used to adjust for it [48].

### Genotype-phenotype Correlation Analysis

To evaluate biological plausibility of our findings, we used the data on *XPF* polymorphism genotypes and *XPF* transcript (mRNA) expression levels both available for 270 lymphoblastoid cell lines by SNPexp online tool (http://app3.titan.uio.no/biotools/help.php?app=snpexp), which provides a convenient and platform-independent way to calculate and visualize the correlation between the HapMap genotypes in a genomic region of interest and gene expression levels [49]. The genotyping data were from the international HapMap phase II release #23 dataset (http://www.hapmap.org) consisting of 3.96 million SNPs that were genotyped using genomic DNA from the 270 individuals from four worldwide populations [CEU: 90 Utah residents with ancestry from northern and western Europe; CHB: 45 unrelated Han Chinese in Beijing; JPT: 45 unrelated Japanese in Tokyo; YRI: 90 Yoruba in Ibadan, Nigeria] [50], [51]. The data of gene expression levels in the same 270 HapMap individuals were from GENEVAR (GENe Expression VARiation, http://www.sanger.ac.uk/resources/software/genevar/) and were detected by using genome-wide expression arrays (47294 transcripts) from EBV-transformed lymphoblastoid cell lines [52]. Student’s *t* test and analysis of variance test were used to evaluate the differences in the relative mRNA expression levels among different genotype groups. All analyses were conducted by using STATA version 11.0 (Stata Corporation, College Station, TX) and SAS version 9.1 (SAS Institute, Cary, NC). All *P* values were two-sided with a significance level of *P*<0.05.

## Results

### Flow of Included Studies

**Figure 2:**
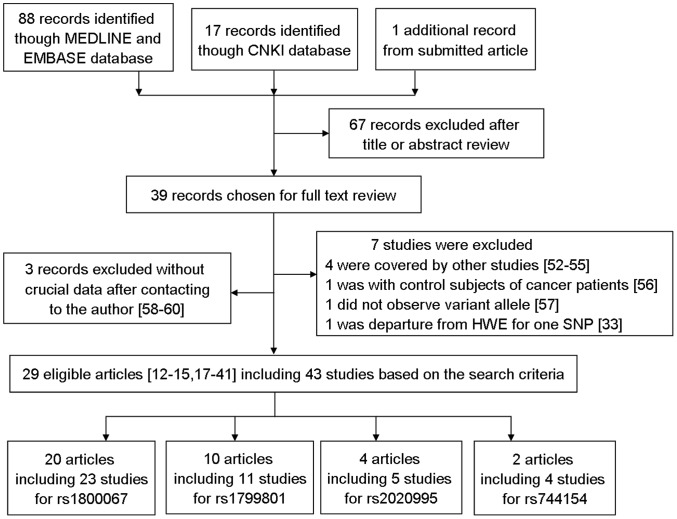
Flow diagram of included studies for this meta-analysis.

As showed in **Figure 2**, a total of 88 published and unduplicated records from the MEDLINE and EMBASE databases, 17 records from the CNKI database and one submitted record were retrieved by using the key words mentioned in the Methods, of which 39 studies examined the association of the commonly studied *XPF* polymorphisms [i.e., rs1800067 (Arg415Gln, exon 8), rs1799801 (Ser835Ser, exon 11), rs2020955 (Ser662Pro, exon10) and rs744154 (intron 1); **Figure 1B**] with cancer risk. Among those 39 publications, four [53]–[56] were excluded because their patient populations were included in other studies [12], [15], [31], one case-control study was excluded because control subjects were of cancer patients [57], one was excluded because no variant allele was observed [58], one study was excluded for departure of the genotype distribution from HWE [33], and three was excluded because of unavailable data to extract ORs and 95% CIs even after having contacted the authors [59]–[61]. The remaining 29 publications of case-control studies contained 43 case-control studies, with a total of 47,639 cancer cases and 51,915 controls of different ethnicities for studying the four polymorphisms.

### Studies Characteristics

**Table 1** lists the essential information for all studies, including first author, year of publication, cancer type, country, ethnicity, study type, source of control, numbers of cases and controls, MAF of controls, statistical power, source of DNA and genotyping methods, grouped by different polymorphisms. For the *XPF*-rs1800067 SNP, the final analysis included nine breast cancer studies [13], [21], [23], [27], [29], [31], [32], four colorectal cancer studies [14], [22], [24], [28], three cancer studies of head and neck [18], [25], [41], two lung cancer studies [15], [20], and five studies of other cancers [12], [19], [26], [30]. Overall, 17 studies used Caucasians, three used African Americans, one used Latinos, and two used mixed ethnic populations. There were 12, nine, one and one studies using PB, HB, PB/HB and FB design, respectively. For the *XPF*-rs1799801 SNP, the final analysis included three prostate cancer studies [19], [34], three bladder cancer studies [33], [35], [39], two breast cancer studies [17], [37] and three studies of other cancers [12], [36], [38]. Among them, six studies used Caucasians, two used African Americans, and three used Asians. Six studies were PB design and five HB design. In addition, there were five and four studies having investigated rs2020955 [27], [30], [33], [34] and rs744154 SNPs [39], [40], respectively.

**Table 1:** Characteristics of studies included in the meta-analysis for an association between *XPF-*rs1800067, rs1799801, rs2020995, rs744154 SNPs and risk of cancers.

First author, Year	Cancer type	Country	Ethnicity	Study type	Source of control	Cases/Controls	MAF of controls	*P* a	Power^b^	Source of DNA	Genotyping methods
***XPF-rs1800067 (Arg415Gln)***											
Yu, submitted	Head neck	USA	Caucasian	Retrospective	HB	1040/1046	0.11	0.485	0.999	Blood	SNPlex, PCR-RFLP, Quantitative PCR
Doherty, 2011	Endometrial	USA	Caucasian	Retrospective	PB	703/714	0.07	0.184	0.925	Blood, Buccal cells	SNPlex, SNaPshot, Quantitative PCR, PCR-RFLP
Gil,2011	Colorectal	Poland	Caucasian	Retrospective	HB	133/98	0.08	0.281	0.533	Lymphocytes	PCR-RFLP
Krupa, 2011	Larynx	Poland	Caucasian	Retrospective	HB	253/253	0.06	0.682	0.876	Lymphocytes	PCR-RFLP
Agalliu, 2010	Prostate	USA	Caucasian	Retrospective	PB	1221/1219	0.09	0.537	1	Lymphocytes	SNPlex
Agalliu, 2010	Prostate	USA	African American	Retrospective	PB	144/81	0.02	0.539	0.489	Lymphocytes	SNPlex
Rajaraman, 2010	Brain	USA	Caucasian	Retrospective	HB	526/471	0.07	0.134	0.809	Blood	Quantitative PCR
Abbasi, 2009	Larynx	Germany	Caucasian	Retrospective	PB	248/647	0.07	0.163	0.747	Buffy coats	Quantitative PCR
Han, 2009	Breast	USA	Caucasian	Prospective	PB	1145/1142	0.07	0.527	0.998	Blood	BeadArray
Joshi, 2009	Colorectal	USA	Caucasian	Retrospective	FB	305/357	–	0.985	0.969	Lymphocytes	Quantitative PCR
Chang, 2008	Lung	USA	Latino	Retrospective	PB	113/299	0.06	0.331	0.609	Blood, Buccal cells	BeadArray
Hung, 2008	Lung	Europe	Caucasian, Asia	Retrospective	PB,HB	2520/2619	0.08	0.002	0.995	Blood	Pooled
McWilliam, 2008	Pancreatic	USA	Caucasian	Retrospective	HB	470/596	0.10	0.003	0.418	Blood	SNPstream, Sequencing
Rajaraman, 2008	Breast	USA	Caucasian	Retrospective	PB	838/1069	–	0.516	0.995	Blood	Quantitative PCR
Smith, 2008	Breast	USA	Caucasian	Retrospective	HB	324/406	0.01	0.342	0.826	Blood	MassArray
Smith, 2008	Breast	USA	African American	Retrospective	HB	53/75	0.06	0.724	0.520	Blood	MassArray
Crew, 2007	Breast	USA	Caucasian	Retrospective	PB	1018/1065	0.09	0.535	0.999	Lymphocytes	FP, Sequenom, Quantitative PCR
Jorgensen, 2007	Breast	USA	Caucasian	Prospective	PB	259/275	0.08	0.671	0.920	Buffy coats	Quantitative PCR
Huang, 2006	Colorectal	USA	Mixed	Retrospective	PB	703/716	0.07	0.313	0.960	Blood	Pooled
Mechanic, 2006	Breast	USA	African American	Retrospective	PB	757/673	0.02	0.034	0.351	Lymphocytes	Quantitative PCR, Sequencing
Mechanic, 2006	Breast	USA	Caucasian	Retrospective	PB	1246/1133	0.07	0.113	0.979	Lymphocytes	Quantitative PCR, Sequencing
Moreno, 2006	Colorectal	Spain	Caucasian	Retrospective	HB	360/323	0.11	0.693	0.969	Blood	APEX microarray
Smith, 2003	Breast	USA	Caucasian	Retrospective	HB	253/268	0.06	0.439	0.799	Blood	PCR-RFLP
***XPF-rs1799801 (Ser835Ser)***											
Doherty, 2011	Endometrial	USA	Caucasian	Retrospective	PB	722/727	0.29	0.896	1	Blood, Buccal cells	SNPlex, SNaPshot, Quantitative PCR, PCR-RFLP
Roberts, 2011	Breast	USA	Caucasian	Retrospective	PB	1063/1913	0.29	0.141	1	Blood, mouthwash samples	MassARRAY
Agalliu, 2010	Prostate	USA	Caucasian	Retrospective	PB	1259/1243	0.29	0.937	1	Lymphocytes	SNPlex
Agalliu, 2010	Prostate	USA	African American	Retrospective	PB	145/79	0.16	0.603	0.806	Lymphocytes	SNPlex
Wang, 2010	Bladder	China	Asian	Retrospective	HB	234/250	0.25	0.412	0.920	Blood	PCR-RFLP
Hooker, 2008	Prostate	USA	African American	Retrospective	HB	252/301	0.13	0.215	0.812	Blood	MassARRAY
Povey, 2007	Melanoma	UK	Caucasian	Retrospective	PB	506/441	0.28	0.041	0.856	Blood	PCR-RFLP
Garcia-Closas, 2006	Bladder	Spain	Caucasian	Retrospective	HB	1091/1019	0.31	0.885	1	leukocytes, mouthwash sample	Pooled
Lee, 2005	Breast	Korea	Asian	Retrospective	HB	386/336	0.23	0.406	0.970	Blood	DASH Hybaid
Matullo, 2005	Bladder	UK	Caucasian	Retrospective	HB	204/213	0.32	0.340	0.865	buffy coats	Pooled
Shen, 2005	Lung	USA	Asian	Retrospective	PB	117/111	0.19	0.231	0.611	Sputum	Quantitative PCR
***XPF-rs2020955 (Ser662Pro)***											
Rajaraman, 2010	Brain	USA	Caucasian	Retrospective	HB	514/444	0.00	0.917	0.589	Blood	Quantitative PCR
Hooker, 2008	Prostate	USA	African American	Retrospective	HB	254/301	0.19	0.689	0.972	Blood	MassARRAY
Garcia-Closas, 2006	Bladder	Spain	Caucasian	Retrospective	HB	1066/1001	0.01	0.859	0.854	leukocytes, mouthwash sample	Pooled
Mechanic, 2006	Breast	USA	African American	Retrospective	PB	752/674	0.20	0.535	0.999	Lymphocytes	Quantitative PCR, Sequencing
Mechanic, 2006	Breast	USA	Caucasian	Retrospective	PB	249/250	0.00	0.568	0.399	Lymphocytes	Quantitative PCR, Sequencing
***XPF-rs744154 (intron)***											
Wang, 2010	Bladder	China	Asian	Retrospective	HB	234/250	0.27	0.004	0.252	Blood	PCR-RFLP
Gaudet, 2009	Breast	Multicenter	Caucasian	Retrospective	PB,HB	25743/29074	0.28	0.542	1	Pooled	Quantitative PCR
Gaudet, 2009	Breast	Multicenter	African	Retrospective	PB,HB	651/571	0.25	0.870	1	Pooled	Quantitative PCR
Gaudet, 2009	Breast	Multicenter	Asian	Retrospective	PB,HB	2700/2104	0.24	0.345	1	Pooled	Quantitative PCR

Abbreviation: SNP, single nucleotide polymorphism; HB, Hospital based; PB, Population based; FB, Family based; RFLP, Restriction fragment length polymorphisms.

a *P* value for dominant genetic model in the associations with *XPF* polymorphisms.

b Statistical power to detect an OR of 1.5 (or 0.67 = 1/1.5).

Almost all of the cases were histopathologically confirmed, except for six studies [13], [15], [20], [34], [36], [40]. Controls were mainly matched with cases by age and/or other variables except for five studies [22], [24], [26]–[28]. All the studies reached 50% power to detect the associations between *XPF* polymorphisms and cancer risk, except for five studies [19], [26], [27], [39]. Blood and lymphocytes were the most common source of DNA, and other sources included buccal cells, buffy coat and mouthwash samples. PCR-based methods were most commonly used in genotyping among these studies.

### Meta-analysis Results

**Table 2** lists the main results of the meta-analysis for the four polymorphisms in the *XPF* gene. Given that the xeroderma pigmentosum (XP) syndromes caused by XP germ-line mutations fit a recessive genetic model, in which heterozygotes are unaffected [62], we tested the hypothesis that the *XPF* polymorphisms were associated with overall cancer risk, assuming a recessive genetic model (i.e., only the variant homozygous genotype was considered the risk genotype).

**Table 2:** Meta-analysis of the associations between *XPF* polymorphisms and cancer risk under the XP recessive genetic model.

Variables	No. of	Cases/Controls	Homozygous model				Recessive model				Power^b^
	studies		OR (95%CI)	*P*	*P*_het_ a	*I^2^*	OR (95%CI)	*P*	*P*_het_ a	*I^2^*	
*rs1800067 (Arg415Gln)*			AA *vs.* GG				AA *vs.* (AG + GG)				
All	23	14632/15545	1.21 (0.73–1.99)	0.467	0.020	45.2	1.20 (0.73–1.98)	0.466	0.022	44.6	0.981
Cancer type											
Breast	9	5893/6106	2.27 (0.81–6.35)	0.119	0.040	54.6	2.26 (0.81–6.29)	0.118	0.041	54.1	0.422
Colorectal	4	1501/1494	0.51 (0.06–4.25)	0.535	0.073	68.8	0.51 (0.06–4.16)	0.532	0.076	68.2	0.679
Head neck	3	1541/1946	1.47 (0.42–5.18)	0.551	0.240	29.9	1.47 (0.41–5.23)	0.550	0.238	30.4	0.651
Others	7	5697/5999	0.84 (0.44–1.63)	0.617	0.222	28.4	0.85 (0.44–1.64)	0.629	0.226	27.8	0.930
Lung	2	2633/2918	0.63 (0.32–1.25)	0.185	0.817	0	0.95 (0.56–1.61)	0.841	0.092	64.8	0.891
Prostate	2	1365/1300	2.57 (0.91–7.23)	0.074	/	/	0.95 (0.77–1.17)	0.607	0.482	0	0.579
Brain	1	526/471	0.70 (0.16–3.15)	0.642	/	/	1.30 (0.92–1.84)	0.134	/	/	0.193
Endometrial	1	703/714	0.63 (0.15–2.64)	0.524	/	/	1.22 (0.91–1.65)	0.184	/	/	0.266
Pancreatic	1	470/596	0.13 (0.01–2.42)	0.172	/	/	0.60 (0.43–0.84)	0.003	/	/	/
Ethnicity											
Caucasian	17	10342/11082	1.46 (0.83–2.54)	0.186	0.043	43.1	1.45 (0.83–2.53)	0.192	0.043	43.1	0.829
African American	3	954/829	2.61(0.11–64.2)	0.557	/	/	2.67 (0.11–65.67)	0.548	/	/	/
Latino	1	113/299	0.92(0.04–22.6)	0.956	/	/	0.88 (0.04–21.68)	0.936	/	/	/
Mixed	2	3223/3335	0.42 (0.12–1.50)	0.182	0.192	41.2	0.43 (0.12–1.57)	0.201	0.186	42.9	0.645
Source of controls											
PB	12	8395/9033	1.13 (0.56–2.28)	0.726	0.079	41.9	1.13 (0.56–2.26)	0.740	0.078	42.0	0.945
HB	9	3412/3536	1.71 (0.66–4.43)	0.268	0.062	50.0	1.71 (0.66–4.40)	0.268	0.063	49.7	0.639
PB/HB	1	2520/2619	0.62 (0.31–1.24)	0.179	/	/	0.64 (0.32–1.28)	0.210	/	/	0.570
Sample size											
<500	12	2915/3678	1.99 (0.72–5.48)	0.181	0.164	33.1	1.98 (0.72–5.43)	0.182	0.168	32.6	0.781
500–1000	5	3527/3643	0.56 (0.23–1.36)	0.199	0.453	0.0	0.54 (0.22–1.33)	0.181	0.461	0.0	0.466
>1000	6	8190/8224	1.23 (0.60–2.51)	0.566	0.016	64.1	1.24 (0.62–2.50)	0.545	0.019	63.1	0.731
***rs1799801 (Ser835Ser)***			**CC ** ***vs.*** ** TT**				**CC ** ***vs.*** ** (CT + TT)**				
All	11	5979/6633	0.91 (0.79–1.04)	0.166	0.783	0.0	0.89 (0.78–1.01)	0.080	0.764	0.0	1
Cancer type											
Prostate	3	1656/1623	1.09 (0.82–1.45)	0.554	0.643	0.0	1.09 (0.83–1.43)	0.540	0.705	0.0	0.994
Bladder	3	1529/1482	0.88 (0.68–1.13)	0.305	0.790	0.0	0.85 (0.66–1.08)	0.179	0.793	0.0	0.989
Breast	2	1449/2249	0.83 (0.63–1.08)	0.155	0.219	33.9	0.85 (0.65–1.09)	0.200	0.297	8.2	0.989
Others	3	1345/1279	0.88 (0.66–1.19)	0.412	0.512	0.0	0.81 (0.61–1.08)	0.157	0.458	0.0	0.959
Melanoma	1	117/111	0.84 (0.52–1.34)	0.462	/	/	1.31 (1.01–1.69)	0.041	/	/	0.298
Lung	1	506/441	1.72 (0.54–5.50)	0.363	/	/	1.39 (0.81–2.39)	0.231	/	/	0.314
Endometrial	1	722/727	0.85 (0.57–1.26)	0.417	/	/	1.01 (0.83–1.25)	0.896	/	/	0.986
Ethnicity											
Caucasian	6	4845/5556	0.88 (0.76–1.02)	0.087	0.792	0.0	**0.87 (0.76**–**1.00)**	**0.049**	0.723	0.0	1
African American	2	397/380	1.70 (0.64–4.51)	0.285	0.992	0.0	1.62 (0.61–4.27)	0.331	0.986	0.0	0.444
Asian	3	737/697	1.07 (0.67–1.71)	0.784	0.408	0.0	0.99 (0.62–1.57)	0.963	0.374	0.0	0.968
Source of controls											
PB	6	3812/4514	0.89 (0.76–1.06)	0.185	0.595	0.0	0.88 (0.75–1.04)	0.131	0.541	0.0	1
HB	5	2167/2119	0.94 (0.74–1.19)	0.598	0.632	0.0	0.90 (0.72–1.13)	0.372	0.645	0.0	0.999
Sample size											
<500	6	1338/1290	1.12 (0.79–1.59)	0.518	0.759	0.0	1.03 (0.73–1.44)	0.877	0.705	0.0	0.993
500–1000	2	1228/1168	0.84 (0.62–1.14)	0.274	0.969	0.0	0.78 (0.58–1.05)	0.097	0.616	0.0	0.921
>1000	3	3413/4175	0.88 (0.75–1.05)	0.153	0.357	3.0	0.90 (0.76–1.06)	0.185	0.388	0.0	1
***rs2020955 (Ser662Pro)***			**CC ** ***vs.*** ** TT**				**CC ** ***vs.*** ** (CT + TT)**				
All	5	2835/2670	1.04 (0.70–1.57)	0.835	0.903	0.0	1.07 (0.72–1.60)	0.729	0.897	0.0	0.968
***rs744154 (intron)***			**GG ** ***vs.*** ** CC**				**GG ** ***vs.*** ** (CG + CC)**				
All	4	29328/31999	0.98 (0.92–1.04)	0.507	0.088	54.1	0.98 (0.92–1.04)	0.547	0.140	45.2	1

Abbreviation: HB, Hospital based; PB, Population based; FB, Family based.

The results were in bold, if the 95% CI excluded 1 or *P*<0.05.

a *P* value for heterogeneity test.

b Statistical power to detect an OR of 1.5 (or 0.67 = 1/1.5).

**Figure 3:**
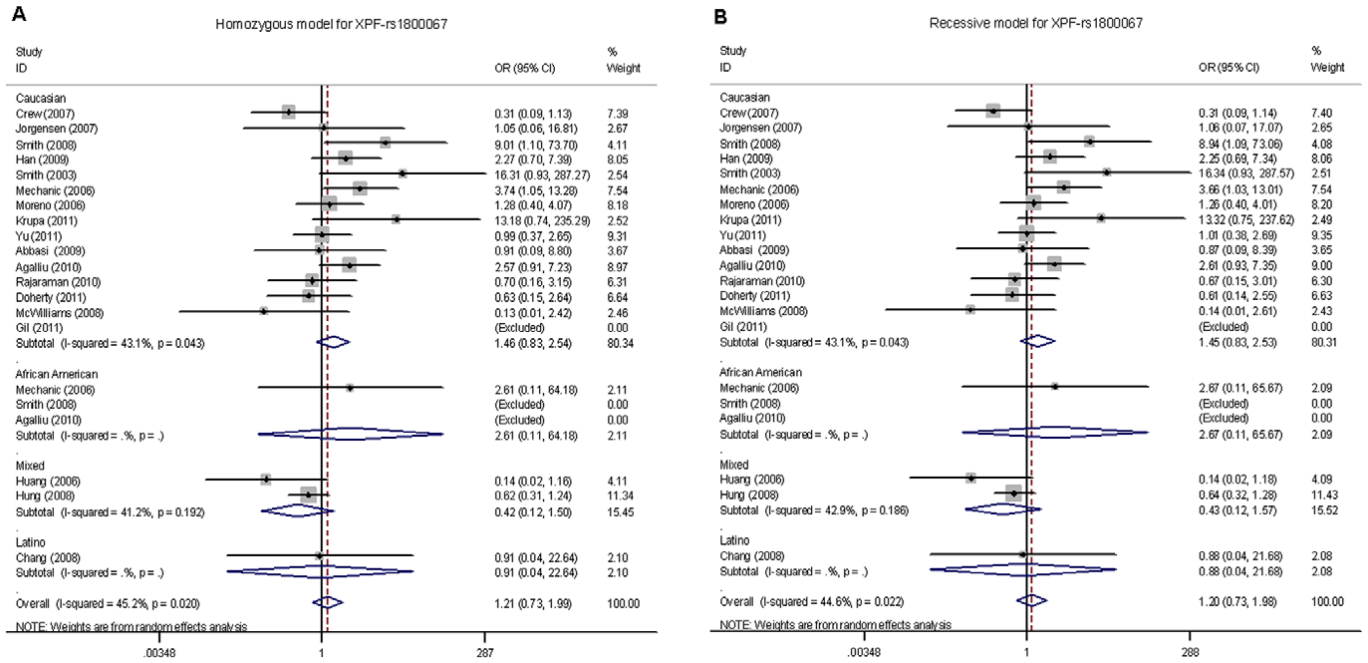
Forest plot of cancer risk associated with the *XPF*-rs1800067 polymorphism stratified by ethnicity. (A) AA *vs.* GG in a homozygous model and (B) AA *vs.* (AG+GG) in a recessive model by the random-effects for each of the 23 published studies. For each study, the estimates of OR and its 95% CI were plotted with a box and a horizontal line. The symbol filled diamond indicates pooled OR and its 95% CI. No significant association between the *XPF*-rs1800067 polymorphism and cancer risk was found.

For the *XPF*-rs1800067 SNP, we obtained genotyping data from 20 publications consisting of 14,632 cancer cases and 15,545 controls. As showed in **Table 2**, when all eligible studies were pooled into the meta-analysis, we found that the *XPF*-rs1800067 polymorphism was not significantly associated with overall cancer risk, with a statistical power of 98% (homozygous model: OR = 1.21, 95% CI = 0.73–1.99, *P* = 0.020 for heterogeneity test, *I^2^* = 45.2%; recessive model: OR = 1.20, 95% CI = 0.73–1.98, *P* = 0.022 for heterogeneity test, *I^2^* = 44.6%). In stratification analyses by cancer type, ethnicity, source of controls or sample size, there was no significant association of *XPF*-rs1800067 SNP with cancer risk in any of the subgroups (**Table 2** **,** **Figure 3A, B**).

**Figure 4:**
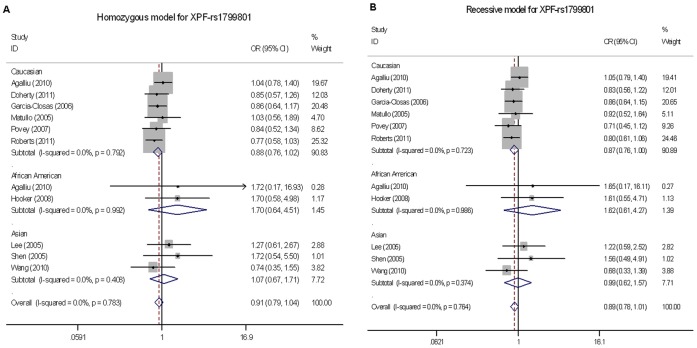
Forest plot of cancer risk associated with the *XPF*-rs1799801 polymorphism stratified by ethnicity. (A) CC *vs.* TT in a homozygous model and (B) CC *vs.* (CT+TT) in a recessive model by the fixed-effects for each of the 11 published studies. For each study, the estimates of OR and its 95% CI were plotted with a box and a horizontal line. The symbol filled diamond indicates pooled OR and its 95% CI. A significant association of the *XPF*-rs1799801 SNP with a borderline cancer risk in Caucasian populations was found (4845 cases and 5556 controls; recessive model: OR = 0.87, 95% CI = 0.76–1.00, *P* = 0.049, *P* = 0.723 for heterogeneity test, *I^2^* = 0).

For the *XPF*-rs1799801 SNP, genotyping data of 5,979 cancer cases and 6,633 controls were obtained from 10 publications. Overall, the *XPF*-rs1799801 polymorphism was not significantly associated with cancer risk (homozygous model: OR = 0.91, 95% CI = 0.79–1.04, *P* = 0.783 for heterogeneity test, *I^2^* = 0; recessive model: OR = 0.89, 95% CI = 0.78–1.01, *P* = 0.764 for heterogeneity test, *I^2^* = 0; **Table 2**). However, in stratification analyses, we found a significant association of the *XPF*-rs1799801 SNP with a reduced cancer risk in Caucasian populations, with a statistical power of 100% (4,845 cases and 5,556 controls; recessive model: OR = 0.87, 95% CI = 0.76–1.00, *P* = 0.049, *P* = 0.723 for heterogeneity test, *I^2^* = 0; **Table 2** **,** **Figure 4A, B**). After stratified by cancer type, source of controls or sample size, no additional significant association of the *XPF*-rs1799801 SNP with overall cancer risk was found in any of the subgroups.

For *XPF*-rs2020955 and rs744154 SNPs, a total of 2,835 cancer cases and 2,670 controls and a total of 29,328 cancer cases and 31,999 controls were included, respectively. No significant association of these two SNPs with cancer risk was found in recessive models (OR = 1.07, 95% CI = 0.72–1.60, *P* = 0.897 for heterogeneity test, *I^2^* = 0%, statistical power = 97%; and OR = 0.98, 95% CI = 0.92–1.04, *P* = 0.140 for heterogeneity test, *I^2^* = 45.2%, statistical power = 100%, respectively; **Table 2**). Because a limited number of published studies for these two polymorphisms were included, no further stratification analysis was performed.

### Heterogeneity and Sensitivity Analyses

Substantial heterogeneities were observed among studies for the association between the *XPF*-rs1800067 polymorphism and cancer risk (homozygous model: χ^2^ = 31.02, df = 17, *P* = 0.020; recessive model: χ^2^ = 30.66, df = 17, *P* = 0.022). Therefore, we used the random-effects model that generated wider CIs. For the other three SNPs of the *XPF* gene (i.e., rs1799801, rs2020955 and rs744154), no heterogeneity was found among studies or in stratification analyses in recessive models (χ^2^ = 6.58, df = 10, *P* = 0.764; χ^2^ = 0.02, df = 1, *P* = 0.897; and χ^2^ = 5.47, df = 3, *P* = 0.140, respectively), and the fixed-effects model was performed. The leave-one-out sensitivity analysis indicated that no single study changed the pooled ORs qualitatively (data not shown).

### Publication Bias

The shapes of the funnel plots seemed symmetrical, and Egger’s test suggested that there was no publication bias for studies of *XPF*-rs1800067, rs1799801, rs2020955 and rs744154 SNPs’ associations with cancer risk in the current meta-analysis [recessive model: *P* = 0.445, 0.205, no value (i.e., Only two studies were included when assumed a recessive genetic model, which caused no value for the Egger’s test) and 0.663, respectively]. These findings indicated that bias from publications, if any, might not have a significant effect on the results of our meta-analysis for the association between the four commonly studied *XPF* polymorphisms and overall cancer risk.

### Correlation Between *XPF*-rs1799801 Genotypes and *XPF* Transcript Expression Levels

**Figure 5:**
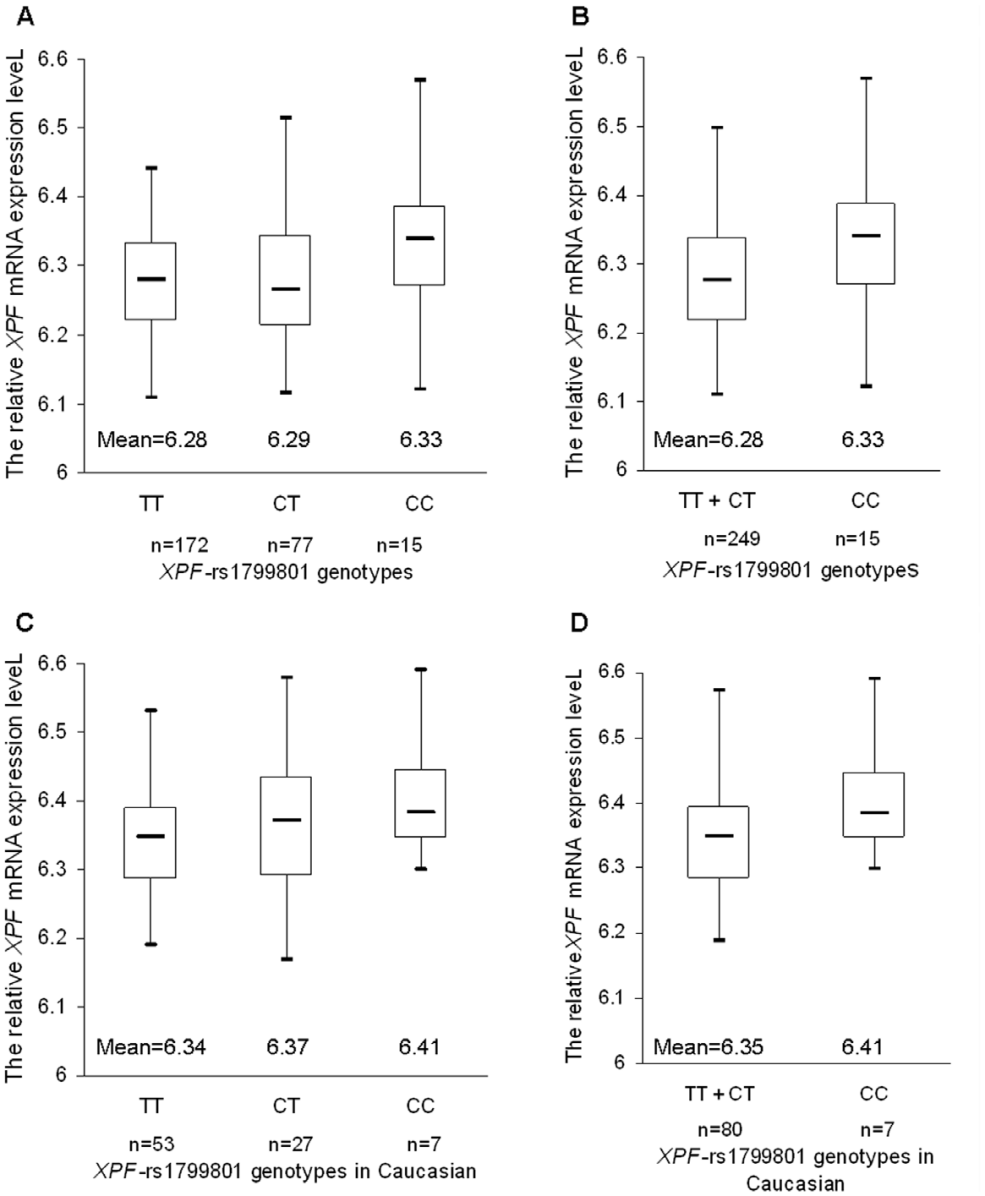
The relative expression levels of *XPF* transcripts by the known *XPF*-rs1799801 genotypes in 270 HapMap subjects. Homozygous variant CC genotype carriers showed a significant increased trend of *XPF* mRNA expression levels in overall populations, compare to (A) wild-type TT genotype ones, and (B) recessive reference TT+CT genotype ones (Student’s *t* test, *P* = 0.032 and 0.046, respectively); but the difference in *XPF* transcript expression levels between the variant CC genotype and (C) wild-type TT genotype, and (D) TT+CT genotypes did not reach statistical significance (Student’s *t* test, *P* = 0.063 and 0.127, respectively).

Given that the *XPF*-rs1799801 SNP, which is located in exon 11, showed a significant association with cancer risk in Caucasian populations, we used the SNPexp online tool to further evaluate biological plausibility underlying the observed association by exploring the correlation between the known *XPF*-rs1799801 genotypes and the relative expression levels of *XPF* transcripts. For the 270 individuals whose genotyping and expression data were available for the analysis, there were 172 TT carriers, 77 CT carriers and 15 CC carriers (**Figure 5A**). Homozygous variant CC genotype carriers had significantly higher *XPF* transcript expression levels than those of wild-type TT carriers and TT+CT carriers (Student’s *t* test, *P* = 0.032 and 0.046, respectively; **Figure 5A, B**). For the 90 Caucasian subjects, 53 TT carriers, 27 CT carriers and seven CC carriers were observed, but the difference in *XPF* transcript expression levels between the variant CC genotype, TT and TT+CT genotypes did not reach statistical significance (Student’s *t* test, *P* = 0.063 and 0.127, respectively; **Figure 5C, D**).

## Discussion

The mechanisms underlying carcinogenesis are multifactorial, and a single genetic variant is usually insufficient to predict risk of cancer, a complex disease phenotype in nature [63]. However, it is likely that suboptimal DNA repair may have a non-specific effect on risk of cancer that originated from DNA damage and subsequent mutation fixation [64]. In this meta-analysis, we summarized all available published data on associations between commonly studied *XPF* polymorphisms and overall cancer risk. Because germ-line mutations in XP genes cause some rare inherited human syndromes, such as XP, cockayne syndrome (CS) and trichothiodystrophy (TTD) following a recessive genetic model [65]–[67], in which mutant homozygotes manifest the disease but the heterozygotes have a normal phenotype [62]. Therefore, we assessed the associations between *XPF* polymorphisms and cancer risk by assuming the XP recessive genetic model.

In this meta-analysis of associations between the four commonly studied *XPF* polymorphisms and cancer risk under the recessive genetic model, we did not find statistical evidence of associations of the *XPF*-rs1800067, rs2020955 and rs744154 SNPs with cancer risk, nor in stratification analyses. One possible explanation is that these variants, especially of rs1800067 and rs744154, are likely to be low-penetrance SNPs with a very weak effect that needs a much larger sample size to detect [63]. Alternatively, these SNPs may not have any effect on cancer risk, given this meta-analysis of pooling all available studies had included a relatively large sample size. There were two obvious differences between our analysis and another recent meta-analysis of the association between the *XPF*-rs1800067 SNP and breast cancer risk by Ding [68]. Firstly, Ding et al. presented only one *XPF* SNP for its association with breast cancer risk, whereas, our analysis included four *XPF* SNPs for their associations with risk of several cancers with a much larger sample size, which provided a more precise assessment of the associations with risk of cancers, including breast, colorectal and other cancers. Secondly, in the present meta-analysis, we also included one more breast cancer study with 1,145 cases and 1,142 controls of Caucasians for the risk association with *XPF*-rs1800067 [13]. Furthermore, the subjects from Crew’s study of 1,018 breast cancer cases and 1,065 controls were predominantly of Caucasians [21], leading to a sample size of more than 2,000 Caucasians added to our new analysis, which increased the weight of Caucasians and study power, although we did not find evidence of any association between the *XPF*-rs1800067 SNP and overall risk of cancers, including breast cancer.

For the *XPF*-rs1799801 SNP, a total of 5,979 cancer cases and 6,633 controls from 10 independent publications were included. Apparently, studies of these Caucasian populations were quite homogenous, compared with those of *XPF*-rs1800067 SNP. Although we did not find any significant association with cancer risk, in the stratification analyses, we did find a significant association between the *XPF*-rs1799801 SNP and cancer risk in Caucasian populations but not in other ethnicities. Further genotype-phenotype correlation analysis showed that homozygous variant CC genotype carriers had significantly increased *XPF* transcript expression levels in all 270 subjects but not in the 90 Caucasians. This inconsistency is likely due to the reduction in the sample size for Caucasian subjects (n = 90), compared with the overall effect by genotypes of all 270 subjects. Another reason could be the heterogeneity of studies included in the analysis of overall risk, such as different weights of ethnicities included in the overall analysis, which may have confounded the results. For example, for the other two ethnicities, especially African American, less than 500 individuals were included with an insufficient statistical power (44.4%) to detect such an association, which might cause a bias in the combined analysis of the association between *XPF*-rs1799801 and cancer risk for all populations.

The *XPF* rs1799801 is highly linked with several other potentially functional SNPs of *XPF*, such as the rs2276466 SNP, which is located at the 3′-untranslated region (UTR) of *XPF*. By using the same HapMap and GENEVAR datasets online, in the overall 270 individuals, there were 175 CC carriers, 77 CG carriers and 16 GG carriers for the *XPF*-rs2276466 SNP. Homozygous variant GG genotype carriers had a significantly higher *XPF* transcript expression levels than that of wild-type CC carriers and CC+CG carriers (Student’s *t* test, *P* = 0.021 and 0.034, respectively). Although the function of the *XPF*-rs2276466 SNP has not been characterized yet, it is well known that variants, located in the 3?-UTR, particularly at a miRNA binding site, may affect mRNA expression levels [69]. Therefore, additional explanation for the correlation of *XPF*-rs1799801 SNP with *XPF* mRNA expression levels may be that some synonymous SNPs appear to act as functional variants in the regulation of gene expression as if they were functional, because of their linkage with other untyped functional SNPs. Additionally, several studies have found that some synonymous SNPs significantly associated with disease phenotypes or traits can be functional by themselves [70], which is because such “silent” polymorphisms may produce a protein product with similar but different structures that may lead to ribosome stalling and alteration of protein folding [71]. Such a hypothesis remains to be tested in future mechanistic studies.

There are several limitations in this meta-analysis, especially for generalization to the general population. First, the quality of the studies included was not optimal. Two studies [14], [15] did not clearly state the ethnicity for genotyping data, and the other two [15], [40] mixed two sources of controls (PB and HB) and did not clearly state the respective genotyping data, which could cause some bias in our estimates. Secondly, obvious heterogeneity across studies for the *XPF*-rs1800067 SNP, which might result from inclusion of imbalanced ethnic groups and types of cancer, existed in overall and some subgroup analyses. Third, for some SNPs (i.e., *XPF*-rs2020955 and rs744154) and a certain subgroup (i.e., African Americans and Asians), the numbers of studies and individual sample sizes were relatively small, having no enough statistical power to detect a weak association. Fourth, our results were based on unadjusted estimates, because not all published studies presented with adjusted estimates or when they did, the estimates were not adjusted by the same potential confounders. A more precise analysis should be conducted, if individual data were available, which would allow for the adjustment by other covariants, including age, ethnicity, smoking status, drinking status, environmental factors, and other lifestyles. Finally, although one unpublished study was included in this meta-analysis, many unpublished data may have not been included in the analysis, potentially causing a bias in our meta-analysis.

Overall, our meta-analysis did not provide statistical evidence for an association between the four commonly studied SNPs of the *XPF* gene and overall risk of several human cancers, but we did find a significant association between the homozygous variant CC genotype of the *XPF*-rs1799801 SNP and a reduced cancer risk in Caucasian populations. Further genotype-phenotype correlation analysis indicated that the *XPF*-rs1799801 homozygous variant CC genotype carriers showed an increased trend of *XPF* expression levels. However, single large, well-designed prospective studies are needed to confirm these findings.
